# Genotypic and Phenotypic Characterization of Axonal Charcot–Marie–Tooth Disease in Childhood: Identification of One Novel and Four Known Mutations

**DOI:** 10.3390/genes16080917

**Published:** 2025-07-30

**Authors:** Rojan İpek, Büşra Eser Çavdartepe, Sevcan Tuğ Bozdoğan, Erman Altunışık, Akçahan Akalın, Mahmut Yaman, Alper Akın, Sefer Kumandaş

**Affiliations:** 1 Department of Pediatric Neurology, Dicle University, Diyarbakır 21280, Türkiye; 2 Department of Medical Genetics, Konya City Hospital, Konya 42020, Türkiye; 3 Department of Medical Genetics, Çukurova University, Adana 01250, Türkiye; 4 Department of Neurology, Adıyaman University Training and Research Hospital, Adıyaman 02040, Türkiye; 5 Department of Pediatric Gentics, Diyarbakır Childrens Diseases Hospital, Diyarbakır 21100, Türkiye; 6 Department of Emergency Medicine, Dicle University, Diyarbakır 21280, Türkiye; 7 Department of Pediatric Cardiology, Dicle University, Diyarbakır 21280, Türkiye; 8 Department of Pediatric Neurology, Pediatric Neurology Clinic, Kayseri 38040, Türkiye

**Keywords:** Charcot–Marie–Tooth disease, axonal neuropathy, pediatric neurology, genetic mutation, gait disturbance

## Abstract

**Background:** Charcot–Marie–Tooth disease (CMT) is a genetically and phenotypically heterogeneous hereditary neuropathy. Axonal CMT type 2 (CMT2) subtypes often exhibit overlapping clinical features, which makes molecular genetic analysis essential for accurate diagnosis and subtype differentiation. **Methods:** This retrospective study included five pediatric patients who presented with gait disturbance, muscle weakness, and foot deformities and were subsequently diagnosed with axonal forms of CMT. Clinical data, electrophysiological studies, neuroimaging, and genetic analyses were evaluated. Whole exome sequencing (WES) was performed in three sporadic cases, while targeted CMT gene panel testing was used for two siblings. Variants were interpreted using ACMG guidelines, supported by public databases (ClinVar, HGMD, and VarSome), and confirmed by Sanger sequencing when available. **Results:** All had absent deep tendon reflexes and distal muscle weakness; three had intellectual disability. One patient was found to carry a novel homozygous frameshift variant (c.2568_2569del) in the *IGHMBP2* gene, consistent with CMT2S. Other variants were identified in the *NEFH* (CMT2CC), *DYNC1H1* (CMT2O), and *MPV17* (CMT2EE) genes. Notably, a previously unreported co-occurrence of *MPV17* mutation and congenital heart disease was observed in one case. **Conclusions:** This study expands the clinical and genetic spectrum of pediatric axonal CMT and highlights the role of early physical examination and molecular diagnostics in detecting rare variants. Identification of a novel *IGHMBP2* variant and unique phenotypic associations provides new insights for future genotype–phenotype correlation studies.

## 1. Introduction

Charcot–Marie–Tooth disease (CMT) was first described by the French neurologists Jean-Martin Charcot and Pierre Marie, together with the English physician Howard Henry Tooth, after whom the disease is named [1]. It is a hereditary neuropathy characterized by slowly progressive, symmetrical muscle weakness and atrophy in the distal extremities, skeletal deformities, sensory deficits, and diminished or absent deep tendon reflexes (DTRs). More than 100 genes have been implicated in CMT, with *PMP22*, *MFN2*, *GJB1*, and *MPZ* among the most commonly affected. In the 1970s, Dyck introduced a classification system that delineated subtypes of CMT based on clinical presentation and electrophysiological findings [2]. However, since the 1990s, it has become increasingly evident that this classification system exhibits substantial heterogeneity due to the wide genetic and phenotypic variability associated with the disease. The age of onset for CMT varies widely depending on the genetic subtype, ranging from infancy to the third or fourth decade of life. The estimated prevalence of CMT ranges from 10 to 28 per 100,000 individuals [3].

CMT is primarily classified into type 1 and type 2 based on nerve conduction studies and histopathological findings. Nevertheless, a significant proportion of axonal CMT cases remain undiagnosed at the molecular level. In this context, our report detailing five pediatric cases with distinct genetic diagnoses—including one harboring a novel—adds meaningful value to the existing literature.

This study aims to enhance diagnostic awareness of axonal CMT by presenting rare cases of children who initially presented with clinical features such as gait disturbances, distal muscle weakness, reduced or absent DTRs, sensory loss, muscle atrophy, and foot deformities and were ultimately diagnosed with different subtypes of axonal CMT.

## 2. Methods

### 2.1. Study Design, Setting, and Ethical Approval

This retrospective descriptive study was conducted in the pediatric neurology outpatient clinics of two tertiary care hospitals in southeastern Türkiye. Medical records of all children evaluated for gait disturbance between January 2022 and January 2023 were screened. Five unrelated patients fulfilled the diagnostic criteria for axonal CMT2 and were included. The study was approved by the Institutional Clinical Research Ethics Committee (Decision No. 2025/132) and was carried out in accordance with the Declaration of Helsinki. Written informed consent for both participation and publication—including clinical photographs—was obtained from the parents or legal guardians of each patient.

### 2.2. Patient Characterization

Demographic data (age, sex), perinatal and developmental history, family history (including degree of parental consanguinity), and presenting complaints were abstracted from the electronic record. A standardized examination captured the following:

- Neurological findings—muscle strength by Medical Research Council (MRC) scale, deep-tendon reflexes (DTRs), sensory deficits, presence of camptodactyly or hyperlaxity.
- Orthopedic features—type of foot deformity (pes cavus, rocker-bottom, pes calcaneovalgus), genu recurvatum, pectus excavatum, scoliosis.
- Systemic/laboratory data—serum creatine kinase (CK), liver enzymes, lactate, and abdominal ultrasonography or echocardiography when clinically indicated.

### 2.3. Neuroimaging

All patients underwent cranial and spinal magnetic resonance imaging (MRI) on a 1.5 T Siemens Magnetom Aera system. Fluid-attenuated inversion recovery and T1/T2 sequences were reviewed for leukomalacia, cortical malformations, or arachnoid cysts.

### 2.4. Electrophysiological Evaluation

Nerve-conduction studies (NCS) and, when indicated, needle electromyography (EMG) were performed using a Nihon–Kohden Neuropack X1 EMG/EP system. Sensory nerve action potential (SNAP), sensory conduction velocity (SCV), compound muscle action potential (CMAP), and motor conduction velocity (MCV) were recorded for sural, tibial, and peroneal nerves under controlled limb temperature (≥32 °C). Demyelinating features were defined as prolonged distal latencies and reduced conduction velocities; axonal involvement was inferred from low-amplitude or absent SNAP/CMAP responses.

### 2.5. Genetic Testing and Bioinformatic Analysis

Peripheral-blood genomic DNA was extracted with the QIAamp DNA Blood Mini Kit (Qiagen, Hilden, Germany). Two complementary next-generation sequencing strategies were used:

1. Whole-Exome Sequencing (WES)—applied to three sporadic cases. Library preparation employed the Twist Human Core Exome Kit (Twist Bioscience, South San Francisco, CA, USA); paired-end (2 × 150 bp) sequencing was performed on an Illumina NovaSeq 6000 platform (Illumina, San Diego, CA, USA), achieving a mean on-target depth > 100×. Reads were aligned to GRCh38 with BWA-MEM; variant calling used the GATK v4 pipeline.
2. Targeted CMT Gene Panel—applied to the two siblings (Cases 4a/4b). A 72-gene hereditary neuropathy panel (TruSight™ Inherited Neuropathy, Illumina) was sequenced on an Illumina MiSeq.

Variant annotation utilized ANNOVAR (v2018Apr16)**,** ClinVar (accessed May 2025)**,** HGMD Professional (v2024.3), and VarSome (accessed July 2025) databases. Pathogenicity was assigned according to the 2015 ACMG/AMP guidelines; criteria met (e.g., PVS1, PM2, PP3) are reported in Table 1. Given the consanguineous background of the families, all identified homozygous variants were further evaluated for clinical relevance. Variant classification was performed in accordance with the American College of Medical Genetics and Genomics (ACMG) guidelines. Annotation and interpretation were supported by population frequency data and clinical databases, including ClinVar, HGMD, and VarSome. Variants of uncertain significance (VUS) and those deemed benign or likely benign were systematically reviewed and documented. No other homozygous variants or VUS variants that could influence the phenotype were detected in any of the cases. All candidate variants, along with parental segregation analyses when DNA was available, were confirmed by Sanger sequencing on an ABI 3500 Genetic Analyzer (Applied Biosystems, Foster City, CA, USA).

## 3. Results

**Figure 1:**
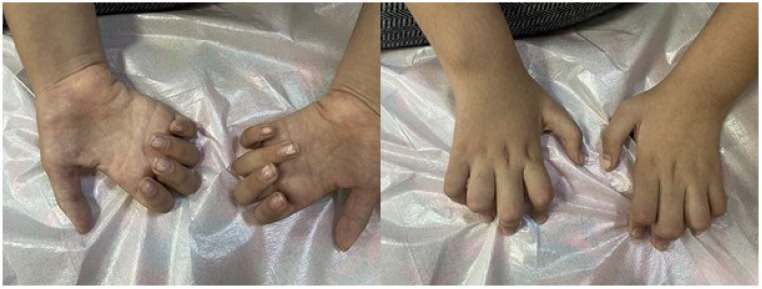
Thenar and hypothenar atrophy (**left photo**) and camptodactyly (**right photo**) (Case 1, girl patient).

**Figure 2:**
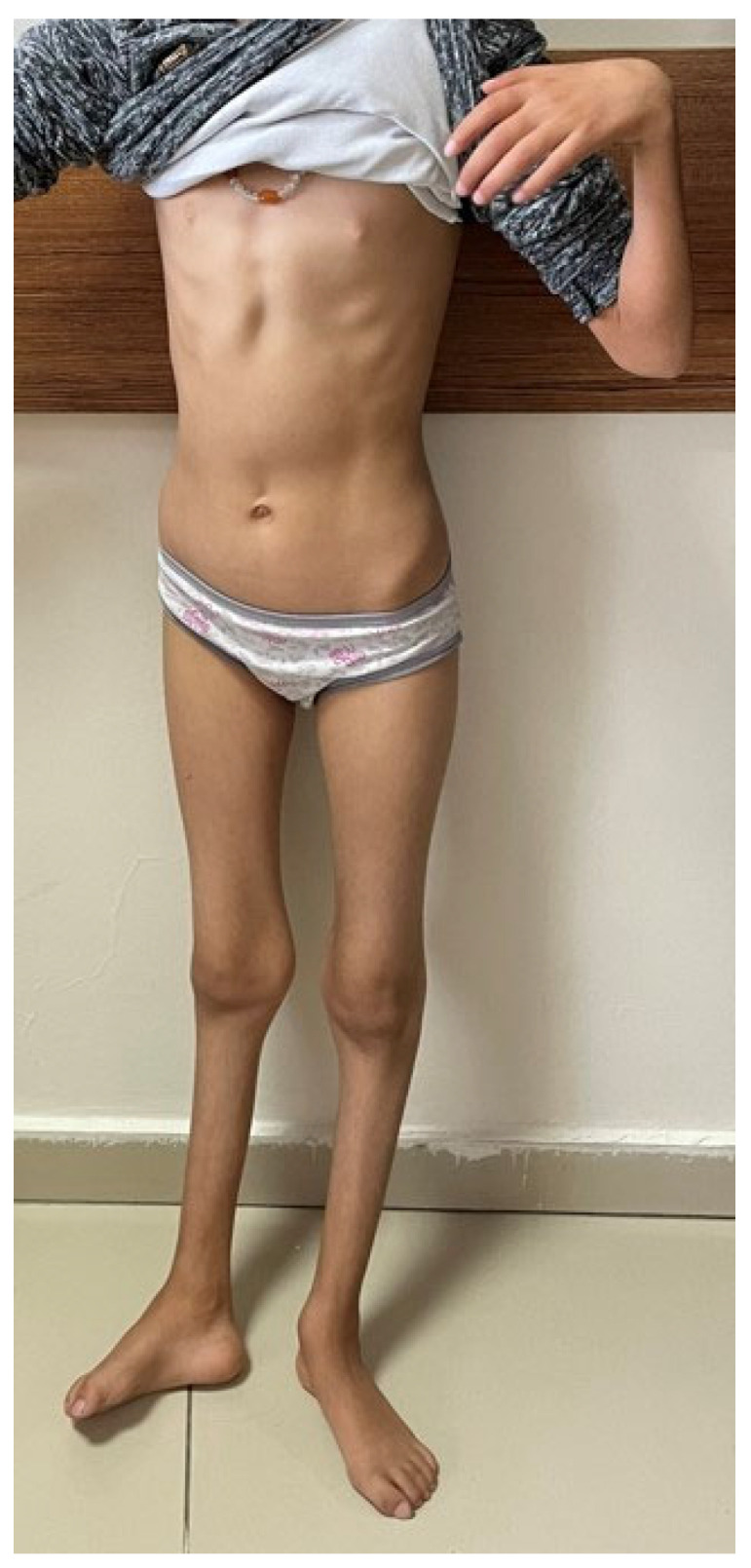
Hyperlaxity, camptodactyly, genu recurvatum, atrophic distal lower extremity, rocker bottom feet, and prominent pes cavus deformity (Case 2, girl patient).

**Figure 3:**
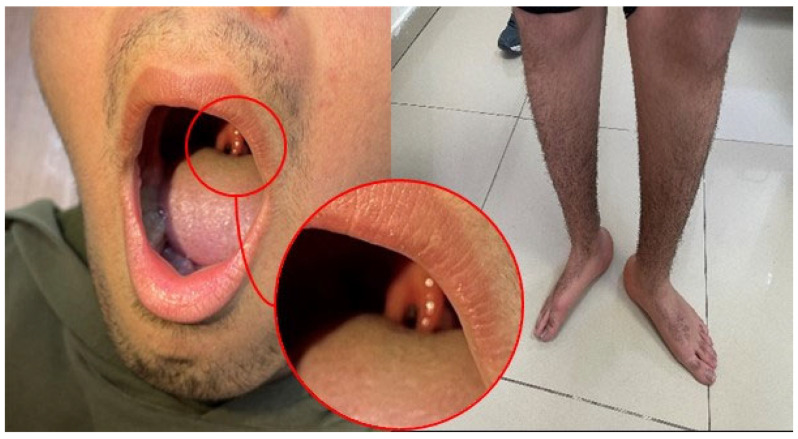
Bifid uvula (**left photo**) and rocker bottom feet and pes calcaneovalgus deformity (**right photo**) (Case 3, boy patient).

**Figure 4:**
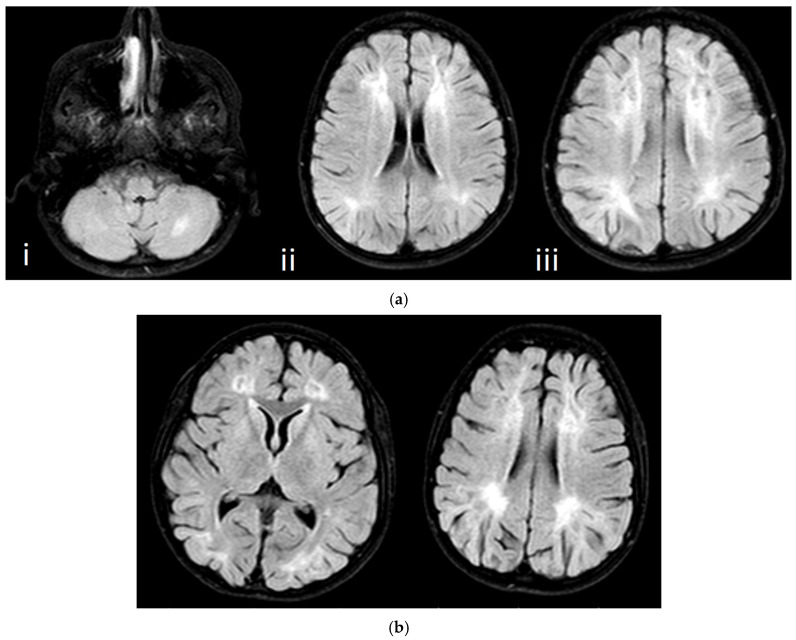
MRI images: (**a**) Case 4a—i. a 13 × 6 mm hyperintense leukomalacic area in the left cerebellum in the fluid-attenuated inversion recovery (FLAIR) sequence, ii. symmetrical hyperintense leukomalacic areas in periventricular deep white matter in the vicinity of the anterior and posterior horns of both lateral ventricles in the FLAIR sequence in cranial MRI examination, iii. symmetrical hyperintense leukomalacic areas in centrum semiovale the FLAIR sequence in cranial MRI examination, and (**b**) Case 4b—symmetrical hyperintense leukomalacic areas in periventricular deep white matter in the vicinity of the anterior and posterior horns of both lateral ventricles in the FLAIR sequence in cranial MRI examination.

Individual clinical details are summarized in Table 2, while representative photographs are provided in Figure 1, Figure 2, Figure 3 and Figure 4.

### 3.1. Case 1

A 15-year-and-9-month-old female was admitted complaining of gait disturbance. Born full term, C/S, 4000 gr. Presenting symptoms were flexion contracture in the right hand at birth, development of camptodactyly in both hands over time, and increased laxity in the bilateral wrist and ankle. The patient’s general and neurological examination findings are summarized in Table 2 and Figure 1. No significant findings were observed in laboratory findings other than CK elevation (Table 2). NCS and cerebral imaging results are included in Table 1. In molecular genetics analysis, ES analysis detected a heterozygous NM_021076.4: c.2434_2436delAAG, p.K812del variant in the *NEFH* gene. The segregation analysis revealed that the mother carried a heterozygous variant, whereas the father was identified as wild type. Since the disease manifests with varying ages at onset, spanning from early childhood to the fourth decade, and exhibits a broad clinical spectrum, this change was interpreted as a variant of uncertain significance (VUS) when evaluated according to the American College of Medical Genetics and Genomics (ACMG) criteria. The variant had a CADD PHRED-scaled score of 16.67. This variant has been described before, and its rs number is given as 1454410518 at VarSome (The Human Genomics Community) [4], but its association with the disease phenotype has not been reported in the literature. The variants were not found in public databases (GnomAD) or reported in the Clinvar and Human Gene Mutation Database (HGMD). Given that axonal type 2C in CMT, associated with the autosomal dominant (AD) inherited *NEFH* gene, is characterized by variable age of onset and clinical severity, the mother carrying the same mutation underwent clinical follow-up.

### 3.2. Case 2

A 9-year-and-9-month-old female was admitted complaining of gait disturbance. Born full term, normal spontaneous vaginal delivery (NSVD), 1500 gr. Presenting symptoms were toe walking and strabismus when started to walk. The patient’s general and neurological examination findings are summarized in Table 2 and Figure 2. No significant findings were observed in laboratory findings other than CK elevation (Table 2). NCS and cerebral imaging results are included in Table 1. In molecular genetics analysis, the ES analysis showed a homozygous NM_002180.3: c.2568_2569del, p.G857Afs*27 variant in the *IGHMBP2* gene. Via segregation analysis, the parents were found to be heterozygous carriers. This variant was interpreted as likely pathogenic according to the ACMG criteria. This variant has not been previously reported in public databases.

### 3.3. Case 3

A 14-year-old male was admitted complaining of gait disturbance. Born full term, NSVD, 4000 gr. Presenting symptoms were deformity in both feet at birth. The patient’s general and neurological examination findings are summarized in Table 2 and Figure 3. No significant findings were observed in laboratory findings other than CK elevation (Table 2). NCS and cerebral imaging results are included in Table 1. In molecular genetics analysis, we identified a heterozygous NM_001376.5: c.2011A > G, p.K671E missense variant detected in the *DYNC1H1* gene. This variant was interpreted as a VUS according to the ACMG criteria. The variant had a CADD PHRED-scaled score of 22.5, indicating a high likelihood of pathogenicity. This variant has been previously associated with spinal muscular atrophy (SMA) (OMIM# 158600) with greater involvement of the lower extremities in the ClinVar database (rs387906742), and also defined in HGMD (CM124332). Parents did not have similar complaints, and their neurological examination was normal. Although segregation analysis was planned, it could not be performed due to the unavailability of parental DNA samples. As the inheritance pattern is autosomal dominant and both parents were reported to be asymptomatic, the variant is presumed to be more likely de novo. However, in the absence of segregation data and functional studies, the pathogenicity of the variant remains uncertain, underscoring the need for careful clinical correlation and follow-up.

### 3.4. Case 4a

A 10-year-and-11-month-old male was admitted complaining of gait disturbance. Born full term, NSVD, 3250 gr. Presenting symptoms were supination of the right foot at 7 years and delayed neuromotor developmental stages. The patient’s general and neurological examination findings are summarized in Table 2. No significant findings were observed in laboratory findings other than liver enzymes, lactate elevation, and urine ketone that was positive and abnormal abdominal USG (Table 2). NCS and cerebral imaging results are included in Table 1 and Figure 4a. In molecular genetics analysis, the CMT panel analysis showed a homozygous NM_002437.5: c.121C > T, p.R41W missense variant in the *MPV17* gene. This change was predicted as likely pathogenic according to the ACMG criteria. This variant has been described before (rs863224072). A CADD PHRED-scaled score of 24.6 supports the potential deleteriousness of this variant. This variant was previously defined in HGMD (CM140649) and associated with mitochondrial DNA depletion syndrome type 6 (OMIM #256810).

### 3.5. Case 4b

An 8-year-and-8-month-old female was admitted complaining of gait disturbance. Born at 36 weeks, NSVD, 2500 gr. Presenting symptoms were frequent falls and gait disturbance that started at 8 years and 6 months old, with normal developmental stages apart from short stature. The patient’s general and neurological examination findings are summarized in Table 2. No significant findings were observed in laboratory findings other than liver enzymes, lactate elevation, and urine ketone was positive and abnormal ECHO (Table 2). NCS and cerebral imaging results are included in Table 1 and Figure 4b. In molecular genetics analysis, the clinical suspect was also the sister of Case 4a. Therefore, Sanger sequencing analysis was conducted on the *MPV17* gene. As a result, the same homozygous NM_002437.5: c.121C > T, p.R41W missense variant was found. The parents of Case 4 were identified as carriers of a heterozygous variant.

In cases where axon loss is widespread and severe, amplitudes and nerve conduction velocities may be recorded as 0 in nerve conduction studies. If all motor and sensory fibers are dysfunctional, amplitudes cannot be recorded, meaning the potentials are 0. Conduction velocities cannot be measured in this case either [5]. As in Case 3, nerve conduction studies in CMT2 cases can sometimes appear normal, especially in the early stages or in milder forms of the disease. Some subtypes (e.g., CMT2D) may have normal or very close results on nerve conduction studies even when the disease progresses [6]. The electrophysiological findings in Case 3 were obtained and represent the current examination of the patient. A peroneal nerve examination was also performed, and the conduction velocity, compound muscle action potential, and conduction latencies of the peroneal nerve were within normal limits.

Abnormal findings are detailed in Table 1 and illustrated in Figure 4.

Complete results appear in Table 3.

## 4. Discussion

The common CMT subtypes are autosomal recessive (AR); indeed, three of our patients exhibited AR inheritance, while two showed autosomal dominant (AD) transmission. CMT1 is the most common subtype of CMT, accounting for approximately two-thirds of all cases. It is commonly referred to as “demyelinating” CMT due to damage to the myelin sheath. CMT2 is a less common subtype of CMT, although it presents with clinical findings similar to those of CMT1. Although symptom onset in CMT2 occurs at a later age compared to CMT1, the clinical manifestations are generally less severe, with predominant motor involvement rather than sensory impairment. Unlike CMT1, which results from damage to the myelin sheath insulating the axons, CMT2 arises from direct damage to the nerve axons themselves. CMT2 is commonly referred to as “axonal” CMT. CMT1 is associated with primary demyelination and motor conduction velocities below 38 m/s, whereas CMT2 is characterized by axonal degeneration with velocities typically above 38 m/s. CMT is a hereditary peripheral neuropathy that affects both motor and sensory nerves. Clinical examination remains pivotal: distal lower-limb weakness is the hallmark, accompanied by motor-predominant symptoms in all of our cases, while sensory involvement was prominent only in Case 1. Over time, the disease manifests as gait instability with frequent falls, pes cavus, atrophy of intrinsic hand muscles, and, later, diminished fine-motor skills. All patients reported longstanding gait disturbances; marked hand-muscle atrophy was particularly evident in Cases 1 and 2. Except for Cases 4a and 4b, disease duration at presentation was under one year.

A high-arched palate, mild creatine kinase (CK) elevation, and pyramidal signs suggestive of myopathy have been linked to *NEFH* (CMT2CC) mutations [7]. Our Case 1 showed similar findings with mildly elevated CK. Rebelo et al. described *NEFH*-mutated cases with normal brain imaging [6]; likewise, cerebral and spinal MRI were unremarkable in our patient, whose electrophysiology indicated axonal neuropathy. Rebelo et al. also noted late-onset manifestations in the patient’s father and grandmother, with the father being able to run until age 48 and the grandmother becoming wheelchair-bound in her mid-seventies. Although the *NEFH* variant in Case 1 is located in the last exon, it is an in-frame deletion of a single lysine residue and does not introduce a premature stop codon. Therefore, the truncating mechanism described by Rebelo et al. may not apply in this context. This variant is listed in dbSNP (rs145441051) and has been reported at low frequency in the GnomAD database. Although the patient’s phenotype is consistent with previously reported cases associated with variants in this gene, the limited predicted structural and functional impact of this specific deletion, along with its presence in population databases, renders its pathogenicity uncertain. Consequently, we acknowledge that the molecular etiology of the patient’s condition cannot be definitively established based on current findings. It remains possible that additional disease-contributing variants undetectable by WES—such as deep intronic, regulatory, or structural variants—may be present, and further investigation may be warranted. [8]. Their electromyography (EMG) revealed myopathic changes and raised CK—features mirrored in Case 1. Interestingly, although the same “possibly pathogenic” variant was detected in Case 1 and her asymptomatic 52-year-old mother, the mother’s only complaint was leg pain; neurological examination demonstrated full strength and preserved deep-tendon reflexes. Given the variable age of onset (early childhood to the fourth decade) and variable severity reported in OMIM #616924, incomplete penetrance is plausible. Segregation analysis was impossible because the mother was lost to follow-up, and nerve-conduction studies (NCS) could not be performed. While Case 1 fits the clinical spectrum of CMT2, larger cohorts and functional studies are required to confirm the variant’s pathogenicity.

CMT2S, caused by biallelic *IGHMBP2* variants, usually presents as a severe, progressive neuropathy in the first decade. WES in Case 2 revealed a novel homozygous frameshift variant c.2568_2569del (p.Gly857Alafs*27) in *IGHMBP2*, leading to loss of function. Symptoms emerged when the patient began walking and evolved into progressive sensorimotor neuropathy without respiratory distress, thereby expanding the phenotypic spectrum of *IGHMBP2*-related disease. Moreover, given the patient’s age, clinical severity, and confirmed biallelic *IGHMBP2* loss-of-function mutation, Case 2 appears to meet the inclusion criteria for the ongoing gene therapy clinical trial NCT05152823. This trial, sponsored by the NIH and investigating AAV9-mediated gene delivery for *IGHMBP2*-related neuropathies, could offer therapeutic benefit in selected cases. Early genetic diagnosis may therefore provide a window of opportunity for novel treatments in severe subtypes like CMT2S [9]. Mandaras et al. ranked CMT2S among the most severe neuropathies [10], which aligns with our clinical and electrophysiological findings. Elevated CK levels, reported by Wagner et al. [11], and upper- and lower-extremity weakness, described by Liu et al. [12], were also seen in our patient.

Heterozygous *DYNC1H1* variants are primarily associated with intellectual disability, cortical malformations, spinal muscular atrophy with lower-extremity predominance (SMA-LED), and the axonal neuropathy subtype CMT2O [13]. SMA-LED follows AD inheritance with proximal lower-extremity weakness, whereas CMT2O manifests as slowly progressive distal weakness and atrophy from early childhood, with normal or near-normal NCS [13]. Our Case 3 displayed pronounced distal lower-limb weakness and atrophy. Cognitive function was normal, though MRI showed ventricular asymmetry and an arachnoid cyst, echoing findings of Tekin et al. [14]. Initial SMN1 testing was negative; needle EMG revealed no denervation. Chan et al. reported normal NCS but neurogenic EMG changes in SMA-LED due to *DYNC1H1* [15]. CK was markedly higher in our patient than in their cohort. A bifid uvula was also noted, but scoliosis did not develop, and the spine MRI was normal. To our knowledge, bifid uvula has not previously been reported in association with *DYNC1H1* variants, making this a potentially novel clinical observation. Consistent with Amabile et al. [16], the phenotypic overlap of *DYNC1H1* disorders complicates diagnosis; thus, broader descriptive categories are now preferred [17]. Given the early onset, slowly progressive distal weakness, lack of neurogenic EMG changes, and preserved cognition, we deemed CMT2O more likely than SMA-LED. Although segregation analysis was initially planned, it could not be performed due to the unavailability of parental DNA samples, and thus, a de novo occurrence remains hypothetical.

CMT2EE typically arises in the first or second decade [18]. One of our patients likewise presented in early childhood. *MPV17* mutations classically cause mitochondrial DNA depletion syndrome 6 (Navajo neurohepatopathy) with infantile, childhood, and classic phenotypes encompassing hepatic dysfunction and neuropathy [19,20]. Blakely et al. described minimal liver-enzyme elevation, elevated cerebrospinal-fluid lactate, and echogenic hepatic ultrasounds [21]. Both Cases 4a and 4b exhibited nonspecific white-matter changes and severe sensorimotor polyneuropathy with axonal and demyelinating features, mirroring literature reports [22]. Case 4a had elevated lactate; Case 4b showed fluctuating liver enzymes and lactate but normal abdominal imaging. The absence of corneal ulceration, nystagmus, hypotonia, dystonia, acute hepatic failure, Reye-like episodes, and hyperbilirubinemia made MTDPS6 less likely (OMIM #256810). Instead, the findings—distal weakness and atrophy, areflexia, lower-limb predominance, abnormal liver enzymes, axonal neuropathy, and white-matter abnormalities—fit CMT2EE [23]. Notably, the *MPV17* variant in our patient coincided with congenital heart disease, representing the first such association in the literature. Additionally, this *MPV17* variant has been curated in the ClinVar database [24]. Notably, alternative amino acid substitutions at the same residue (Arg41) have previously been associated with neuropathy, as reported in two independent studies, further supporting its potential pathogenic role [18,22].

### Limitations

Study limitations include the small cohort size, the single-center, cross-sectional design, and limited resources (absence of nerve- or muscle-biopsy capability and comprehensive genetic panels). Variants of uncertain significance (VUS) detected by next-generation sequencing could not always be confirmed by Sanger sequencing, and follow-up was disrupted by the February 2023 Türkiye earthquake. Larger studies with extended follow-up and additional functional assays are required to elucidate variant pathogenicity and long-term outcomes.

## 5. Conclusions

This study characterizes the clinical, electrophysiological, and molecular spectrum of axonal CMT in childhood through five distinct cases and introduces a novel variant in the *IGHMBP2* gene to the scientific literature. The identified homozygous deletion likely leads to loss of function and expands the known phenotypic range of the CMT2S subtype. Given the clinical similarity across CMT2 subtypes, our findings underscore the critical role of molecular genetic testing in differential diagnosis. The observed genotype–phenotype correlations in this series facilitate the recognition of rare variants and support early diagnosis, genetic counseling, and tailored follow-up in pediatric hereditary neuropathies. Furthermore, this report presents the first documented co-occurrence of the *MPV17* mutation with congenital heart disease, offering a novel perspective that may prompt future investigations. Studies with larger cohorts are warranted to further define these emerging genotype–phenotype relationships.

## Figures and Tables

**Table 1:** Genetic, neurophysiological and imaging findings of all patients.

Case	1	2	3	4a	4b
Nerve conduction study	Sensorimotor polyneuropathy consistent with axonal degeneration	Severe sensorimotor polyneuropathy with axonal degeneration and demyelination	Normal	Severe sensorimotor polyneuropathy with axonal degeneration and demyelination	Severe sensorimotor polyneuropathy with axonal degeneration and demyelination
MRI	Brain: Normal Spine: Normal	Brain: Normal Spine: Normal	Brain: Abnormal Spine: Normal	Brain: Abnormal Spine: Normal (Figure 4a)	Brain: Abnormal Spine: Normal (Figure 4b)
Genetic test	*NEFH* (NM_021076.4)	*IGHMBP2* (NM_002180.3)	*DYNC1H1* (NM_001376.5)	*MPV17* *(NM_002437.5)*	
Clinical Phenotype	CMT2CC	CMT2S	CMT2O	CMT2EE	
Protein change	p.K812del	p.G857Afs*27	p.K671E	p.R41W	
ACMG Classification	VUS (PM4, PM2)	LP (PVS1, PM2)	VUS (PM2, PP2)	LP (PM2, PM5, PP3, PP2, PP5)	
HGMD Variant	c.2434_2436del; p.(Lys812del)	c.2568_2569del; p.(G857Afs*27)	c.2011A > G; p.(K671E)	c.121C > T; p.(R41W)	
Zygosity	Heterozygous	Homozygous	Heterozygous	Homozygous	

LP: Likely Pathogenic, MRI: Magnetic resonance image, VUS: Variant of uncertain significance, HGMD Variant: Human Gene Mutation Database Variant.

**Table 2:** Demographic data and clinical findings of all patients.

Case	1	2	3	4a	4b
Age at diagnosis (years)	15^9/12^	9^9/12^	14	10^11/12^	8^8/12^
Onset age	At birth	12 months old	At birth	7 years old	8 years 6 months old
Gender	Female	Female	Male	Male	Female
Parental consanguinity	First-degree cousins	First-degree cousins	Third-degree cousins	First-degree cousins	First-degree cousins
Cognitive function	Normal	Normal	Normal	Moderate intellectual disability	Mild intellectual disability
High-arched palate	(+)	(−)	(−)	(−)	(−)
Muscle strength	Upper extremity distal: 4/5 (mild) Lower extremity distal: 4/5 (mild)	Upper extremity distal: 4/5 (mild) Lower extremity distal: 4/5 (mild)	Upper extremity distal: 4/5 (mild) Lower extremity distal: 4/5 (mild)	Upper extremity distal: 4/5 Lower extremity distal: 3–4/5 (mild-moderate)	Upper extremity distal: 4/5 (mild) Lower extremity distal: 4/5 (mild)
Muscle atrophy	Lower extremity distal, upper distal, and thenar and hypothenar (Figure 1)	Lower extremity distal, thenar, and hypothenar	Lower extremity distal	Lower extremity distal, thenar, and hypothenar atrophy	Lower extremity distal
DTR	Absent	Absent	Absent	Absent	Absent
Hyperlaxity	(+)	(+)	(−)	(+)	(−)
Camptodactyly	(+)	(+)	(−)	(+)	(−)
Foot deformity	Pes cavus, rocker bottom feet	Pes cavus deformity, rocker bottom feet (Figure 2)	Pes calcaneovalgus deformity, rocker bottom feet (Figure 3)	Pes cavus deformity, contractures in the ankle	Pes cavus deformity, contractures in the ankle
Others	Hypoesthesia Pectus excavatum and scoliosis	Small hands and feet, weak grip Genu recurvatum prominent (Figure 2)	Bifid uvula (Figure 3)	Moderate liver enzyme elevation Nasal speech	Moderate liver enzyme elevation Exhibiting fluctuating
				ALT: 66 (5–40 U/L) AST: 46 (5–40 U/L) NT-ProBNP: 13 pg/mL Lactate: 3.2 (0.7–2 mmol/L) Urine ketone: +2 Slightly coarse granular appearance in the liver parenchyma on abdominal USG ECHO: Normal ECG: Normal	ALT: 53 (5–40 U/L) AST: 79 (5–40 U/L) NT-ProBNP: 15 pg/mL Lactate: NA Urine ketone: +3 ECHO: Bicuspid aorta, ascending aortic dilatation ECG: Normal
CK (29–200 U/L)	315 U/L	568 U/L	632 U/L	54 U/L	33 U/L

ALT: Alanine aminotransferase, AST: Aspartate aminotransferase, CK: Creatine kinase, ECHO: echocardiography, ECG: electrocardiography, DTR: Deep tendon reflex, USG: Ultrasonography, N/A: not available.

**Table 3:** SNAP, CMAP, and nerve conduction velocity values of the right sural and tibial nerves of the cases.

	R Sural Nerve		R Tibial Nerve		R Peroneal Nerve	
	SNAP (µV) (Reference ≥ 4)	SCV (m/s) (Reference ≥ 39)	CMAP (mV) (Reference ≥ 3)	MCV (m/s) (Reference ≥ 39)	CMAP (mV) (Reference ≥ 2.5)	MCV (m/s) (Reference ≥ 39)
Case 1	0.0	0.0	0.0	0.0	0.0	0.0
Case 2	0.0	0.0	0.0	0.0	0.0	0.0
Case 3	30.7	40.3	4.3	42.9	3.8	43.8
Case 4a	2.2	36.3	0.0	0.0	0.0	0.0
Case 4b	1.8	34.8	0.0	0.0	0.0	0.0

CMAP: compound muscle action potential, MCV: motor conduction velocity, SCV: sensorial conduction velocity, SNAP: sensory nerve action potential.

## Data Availability

The original contributions presented in this study are included in the article. Further inquiries can be directed to the corresponding author.
